# Seed micromorphology of *Orchis* Tourn. ex L. (Orchidaceae) and allied genera growing in Edirne province, Turkey

**DOI:** 10.3897/phytokeys.68.8746

**Published:** 2016-08-02

**Authors:** Necmettin Güler

**Affiliations:** 1 Department of Biology, Faculty of Sciences, Trakya University, 22030 Edirne, Turkey

**Keywords:** Anacamptis, Neotinea, Orchis, Orchidaceae, seed micromorphology

## Abstract

In this study, the seed micromorphologies of eight taxa of *Anacamptis*, *Neotinea* and *Orchis* growing around Edirne province (Turkey) were investigated using light microscopy and scanning electron microscopy (SEM). Slides prepared with glycerin jelly were used for measurements under the light microscope and fine details of seed testae characteristics were observed with SEM. Seeds of the investigated orchid taxa are fusiform shaped and of different shades of brown. Their lengths and widths are different among the taxa and range between 0.263–0.640 mm and 0.118–0.208 mm, respectively. Testa surfaces of Orchis mascula subsp. mascula, Orchis purpurea subsp. purpurea and Orchis simia subsp. simia, are smooth while those of *Anacamptis coriophora*, Anacamptis laxiflora subsp. laxiflora, Anacamptis morio subsp. morio, *Anacamptis papilionacea* and Neotinea tridentata subsp. tridentata are reticulate. An identification key based on seed morphologies and sizes is suggested for the first time, including testae structures of orchids growing in Edirne province. The overall results of the study showed that morphological structures of orchid’s seeds could be used as diagnostic characters in identification.

## Introduction

Orchidaceae are one of the most diversified and evolved families in the flowering plants (Cribb and Govaerts 2005). According to a recent survey (Govaerts et al. 2016) the number of the accepted species currently amounts to 24.000 but might reach 30.000, in view of the ever accelerating rate of new species descriptions every year (Tyteca and Klein 2008). The systematics have undergone many changes along the last few decades (Gamarra et al. 2010). The latter taxonomic proposals were published by Dressler (1993) and Szlachetko (1995). In the subfamily Orchidoideae, Dressler (1993) divided the tribe Orchideae into two subtribes: Orchidinae with 34 genera and 370 species, and Habenariinae with 23 genera and 930 species (Gamarra et al. 2010). The genus *Orchis* Tourn. ex L. and allied genera *Anacamptis* Rich. and *Neotinea* Rchb.f. are some of the most controversial groups belonging to the tribe Orchideae (Orchidaceae). The original genus *Orchis* s.l. used to include more than 1,300 taxa and in its broad concept, had a complex taxonomic history (Vermeulen 1972, Klein 1989, 2004, Bateman et al. 1997, 2003, Buttler 2001, Szlachetko 2002, Baumann and Lorenz 2006, Kretzschmar et al. 2007, Tyteca and Klein 2008, Delforge 2009). Since *Orchis* has been proven to be polyphyletic, several species were separated into distinct genera (Bateman et al. 1997). Also, in many guides and floras (see Tutin et al. 1980, Sezik 1984, Renz and Taubenheim 1984, Buttler 1986, Kreutz 1998, Delforge 2006, Buttler 2007) the number of *Orchis* taxa varies considerably, including species that previously belong to other genera, such as *Aceras* R.Br., *Anacamptis* Rich, *Dactylorhiza* Neck. ex Nevski, *Neotinea* Rchb.f. and *Vermeulenia* Á.Löve & D.Löve (Gamarra et al. 2012). Recently, molecular analyses have changed the taxonomy of several species in the genus *Orchis* (Bateman et al. 1997, 2003, Pridgeon et al. 1997). The genera *Anacamptis* and *Neotinea* were traditionally considered each as a monotypic genus, represented by *Anacamptis pyramidalis* (L.) Rich. and *Neotinea maculata* (Desf.) Stearn respectively. Afterwards, the molecular analyses published by Pridgeon et al. (1997) and Bateman et al. (1997) confirmed the polyphyletic status of *Orchis* s.l., and many species were placed into the expanded genera *Anacamptis* and *Neotinea* (Gamarra et al. 2012), such as Anacamptis morio (L.) R.M.Bateman, Pridgeon & M.W.Chase subsp. morio, Anacamptis laxiflora (Lam.) R.M.Bateman, Pridgeon & M.W.Chase subsp. laxiflora, *Anacamptis coriophora* (L.) R.M.Bateman, Pridgeon & M.W.Chase, *Anacamptis papilionacea* (L.) R.M.Bateman, Pridgeon & M.W.Chase and *Neotinea tridentata* (Scop.) R.M.Bateman, Pridgeon & M.W.Chase, etc. In the molecular phylogenetic analyses published by Bateman et al. (1997) and Pridgeon et al. (1997), some *Orchis* species were nested in *Anacamptis* and *Neotinea*. However, based on either morphological or molecular data, the (old) genus *Orchis* has been split into three genera: *Herorchis* D.Tyteca & E.Klein, *Androrchis* D.Tyteca & E.Klein and *Odontorchis* D.Tyteca & E.Klein (see Tyteca and Klein 2008). According to these authors, *Neotinea* and *Anacamptis* returned to their former monotypic position with the species *Neotinea maculata* and *Anacamptis pyramidalis* respectively. The genera *Herorchis* and *Odontorchis* included the rest of the species of *Anacamptis* and *Neotinea* cited respectively, by Kretzschmar et al. (2007), and the genus *Androrchis* contained all the species of the genus *Orchis*, except the group with an anthropomorphic labellum, which is retained in *Orchis* (including *Aceras*). Later, Tyteca and Klein (2008) adopted the enlarged genera *Anacamptis* and *Neotinea* sensu Bateman et al. (1997, 2003), but reaffirmed the segregated genus *Androrchis* (Gamarra et al. 2012). Delforge (2009) published a new classification of *Orchis* s.l. and accepts the taxonomical position of *Orchis* and *Neotinea* sensu Bateman et al. (1997, 2003); however, he did not support the expanded genus *Anacamptis*, considering this genus as monotypic (*Anacamptis pyramidalis*), and segregating the rest of the species into the genera *Herorchis*, *Vermeulenia*, *Anteriorchis* E.Klein & Strack and the new genus *Paludorchis* P.Delforge (Gamarra et al. 2012). In this study, we have chosen the species delimitation of Bateman et al (1997), because it requires the fewest change in nomenclature.

According to Kretzschmar et al. (2007), the genus *Anacamptis* has three part lip, but undivided middle lob, at base, in front of the spur entrance are two raised disks or longitudinal ridges; bracts from at least half as long to (mainly) longer than the ovary. The genera *Orchis* and *Neotinea* have three part lip with +/- divided middle lob, without raised disks or ridges at the base; bracts either clearly shorter or at most as long as the ovary. The genus *Orchis* differs from *Neotinea* with uniform, round or trapezoid stigmatic cavity, longish column and without genuine winter rosette.

The distribution area of the genus *Anacamptis* reaches to the Atlantic in the west and to the Hebrides and southern Scandinavia in the north. It includes the North African mountains in its southwest border, whereas other parts of North Africa and the Canaries remain blank, although it penetrates along the Levant considerably further to the south. The genus in the east reaches to Lake Balchaš in central Asia and its representatives are also found on all the larger islands of the Mediterranean. The ecological demands of the different species are various, but all commonly prefer to settle within biotopes that have seasonal changes, really humid winters, which temporarily become very dry in summer (Delforge 2006, Kretzschmar et al. 2007, Govaerts et al. 2016). The genus *Anacamptis* have 11 accepted species and 20 subspecies (Kretzschmar et al. 2007, Govaerts et al. 2016).

The genus *Orchis* (Orchidaceae, Orchidinae) is limited in its distribution exclusively to the northern hemisphere. Its mainly distribution area is Mediterranean Basin where the maximum density of species is reached; however, other part of Europe are also settled to great extent. In addition the genus with some species, divert out of its main range and reaches northwards to Scandinavia, whilst in an easterly direction to Mongolia and reaches last Lake Baikal. On the north coast of Africa the eastern part is blank to great extent due to the absence of suitable biotopes; however, areas of Asia Minor and further on to Iraq and Iran are included. The ecological demands of the different species are various (Delforge 2006, Kretzschmar et al. 2007, Govaerts et al. 2016). The genus *Orchis* have 21 accepted species and 16 subspecies (Kretzschmar et al. 2007, Govaerts et al. 2016).

The genus *Neotinea* is limited to Europe, Asia Minor, the Caucasus and the north-west coastal regions of North Africa. The ecological demands of the different species are various (Delforge 2006, Kretzschmar et al. 2007, Govaerts et al. 2016). The genus *Neotinea* comprises four accepted species and two subspecies (Kretzschmar et al. 2007, Govaerts et al. 2016).

Seed morphology is one of the important taxonomic characters of orchids. Beer (1863) published the first study about the seed morphology in Orchidaceae, while, the taxonomic importance of the seed characteristics was first pointed out by Clifford and Smith (1969). Arditti et al. (1979) established the methodology for quantitative analyses, related to the sizes and volumes of seeds and embryos. Orchid seeds are characterized by minute and consist of an elliptical embryo enclosed within a generally transparent and often fusiform testa. Testae and embryos of different genera and species may vary in size, shape, color or the ratios between their volumes. The walls of testa cells can be smooth or reticulate and when reticulation is present, its patterns may be distinctive (Arditti 1967, Arditti et al. 1979, 1980, Healey et al. 1980, Chase and Pippen 1988).

The rather small sizes of seeds make them difficult to study their details and to compare some features with only light microscopy. Therefore, making comparisons and determining details that could be used as taxonomical characters without SEM techniques appear to be a challenging task (Arditti et al. 1979). However, if some characters are investigated only by SEM, then this may lead to obtaining of some wrong data. Therefore, relying on the use of both techniques, light microscopy and SEM, complementary to each other will be a better option for a researcher to get a clear picture of the studied question.

Most of the studies performed on orchid seeds were based on tropical orchids whereas the non-tropical species were generally neglected (Arditti 1967, Arditti et al. 1979, 1980, Healey et al. 1980, Chase and Pippen 1987, 1988, Rasmussen and Whigham 1993, Kurzweil 1993, Molvray and Kores 1995, Swamy et al. 2004, 2007, Gamarra et al. 2007, 2010, 2012, Chaudhary et al 2014, Galán Cela et al. 2014).

Several authors published different papers about seed morphology in the genera of *Orchis*, *Anacamptis* and *Neotinea*. Wildhaber (1972) initiated the morphological study of the seeds in the genera *Orchis* and *Neotinea* using light microscopy to obtain a key for the species based principally on the morphology and length of the seeds. Barthlott (1976) confirmed the taxonomic value of the periclinal walls in the genera *Orchis* and *Neotinea*. Ziegler (1981) recognized the characteristic seeds of the genus *Orchis* as *Orchis*-type. Tohda (1983) analyzed the differences in the sculpturing of the testa seeds in some *Orchis* species using SEM images and recognized three groups, two with slanting stripes and one with smooth periclinal walls. Mrkvicka (1994) analyzed quantitative and qualitative data of European *Orchis* using light microscopy, revealing a high diversity in the seed coat micromorphology. Molvray and Kores (1995) provided data on the number of testa cells in *Orchis spectabilis* (L.) Raf. Arditti and Ghani (2000) reviewed the purely numerical and physical characteristics of orchid seeds and their biological implications; among of them *Anacamptis collina* (Banks & Sol. ex Russell) R.M.Bateman, Pridgeon & M.W.Chase (as *Orchis collina* Banks & Sol.), *Anacamptis coriophora* (as *Orchis coriophora*), *Anacamptis morio* (as *Orchis morio*), Anacamptis morio subsp. longicornu (Poir.) H.Kretzschmar, Eccarius & H.Dietr. (as *Orchis longicornu*), *Orchis mascula*, *Orchis purpurea* and *Orchis simia*. Gamarra et al. (2007) analyzed the morphology of the seed and of the anticlinal and periclinal walls using SEM in the genus *Neotinea*. Gamarra et al. (2012) analyzed seeds of 24 taxa belonging to the genera *Anacamptis* and *Orchis*.

Few studies exist on seed morphology of Turkish orchids. One of them was performed by Olgun and Aybeke (1996) on Edirne *Ophrys* L. species using SEM. There are also light microscopy studies on *Ophrys* species (see Aybeke 1997) and *Orchis* species (see Güler 1997) in Edirne Province. The present study aimed to reveal the relationship between *Orchis* and allied genera *Anacamptis* and *Neotinea* species growing naturally in Edirne region and to contribute to species classification based on seed measurement and morphological data.

## Materials and methods

We analyzed seeds of eight taxa belonging to the genera *Orchis*, *Anacamptis* and *Neotinea*. The study material consisting of specimens of eight orchid taxa were collected from the region within Edirne provincial borders in 1995 and 1996 and are kept in EDTU Herbarium. A list of voucher specimens and localities is given in the Table 1. Fresh seeds were dried and stored in small paper envelopes. The identification of the specimens was performed according to local flora and monographs (Tutin et al. 1980, Sezik 1984, Renz and Taubenheim 1984, Buttler 1986, Kreutz 1998, Delforge 2006). The seeds obtained from mature and opened fruits were used for seed morphology investigations. For this purpose, permanent slides of seeds were prepared with glycerin jelly solution on a heating plate (Ozban and Ozmutlu 1994) and the slides were investigated under a light microscope for morphological evaluations. The seeds were measured and then photographed. The color of the seeds were observed and described in annotated subjective terms with the help of optical microscope (Gamarra et al 2012, Chaudhary et al 2014, Galán Cela 2014). The specimens used for SEM were dried and examined for fine structure details.

**Table 1.** The locality and EDTU code details of the studied orchid taxa.

Species	EDTU	Source Locality	Collectors	Date Received
*Anacamptis coriophora*	6075	Kesan, Yayla village	N. Güler	02.06.1995
Anacamptis laxiflora subsp. laxiflora	6074	Kesan, Mecidiye village	N.Güler & M.Aybeke	06.05.1995
Anacamptis morio subsp. morio	6056	Kesan, Yerlisu village	N.Güler & M.Aybeke	22.04.1995
Anacamptis morio subsp. morio	6058	Kesan, Camlica village	N.Güler & M.Aybeke	06.05.1995
Anacamptis morio subsp. morio	6059	Kesan, Camlica-Gökcetepe villages	N.Güler & M.Aybeke	06.05.1995
Anacamptis morio subsp. morio	6062	Kesan, Mecidiye village	N.Güler & M.Aybeke	06.05.1995
Anacamptis morio subsp. morio	6063	Kesan, Yayla village	N.Güler & M.Aybeke	07.05.1995
Anacamptis morio subsp. morio	6065	Enez, Haskoy village	N.Güler	09.05.1995
Anacamptis morio subsp. morio	6067	Lalapasa, Hanliyenice village	N.Güler	16.05.1995
Anacamptis morio subsp. morio	6265	Enez, Abdürrahim village	N.Güler & M.Kirec	02.05.1996
Anacamptis morio subsp. morio	6267	Kesan, Kizkapan village	N.Güler & M.Aybeke	11.05.1996
*Anacamptis papilionacea*	6079	Kesan, Yayla village	N.Güler	02.06.1995
Orchis mascula subsp. mascula	6132	Enez, Candir village	N.Güler & M.Kirec	02.05.1996
Orchis purpurea subsp. purpurea	6119	Uzunköprü, Turnaci village	N.Güler & M.Aybeke	27.05.1995
Orchis purpurea subsp. purpurea	6103	Hasanağa village	N.Güler	25.04.1995
Orchis purpurea subsp. purpurea	6110	Kesan, Suluca village	N.Güler & M.Aybeke	09.05.1995
Orchis purpurea subsp. purpurea	6116	Lalapasa, Dogankoy village	N.Güler	19.05.1995
Orchis simia subsp. simia	6080	Kesan, Yerlisu village	N.Güler & M.Aybeke	15.04.1995
Neotinea tridentata subsp. tridentata	6136	Kesan, Yayla village	N.Güler & M.Aybeke	11.05.1996
Neotinea tridentata subsp. tridentata	6120	B.Ismailce village	N.Güler	19.05.1995
Neotinea tridentata subsp. tridentata	6092	Kesan, Kizkapan village	N.Güler & M.Aybeke	07.05.1995

The terminology and methods were adopted from those of Arditti (1967), Arditti et al. (1979, 1980), Healey et al. (1980), Chase and Pippen (1987, 1988), Kurzweil (1993), Molvray and Kores (1995) and Arditti and Ghani (2000). Measurements of seed embryos for morphometric data were taken using an Olympus BH2 light microscope equipped with a micrometric ocular. Statistical analyses were performed by NCSS 2013 (Version 9.0.5) for Windows. Seed and testa volumes were calculated using the formulations in Arditti et al. (1979). Since all seeds studied were fusiform, closely approximating two cones joined at their bases, their volumes were calculated using the formula: V_t_ = 2[(^W^/_2_)^2^(½ L)(1.047)] where w is the seed width, L is the seed length, and 1.047 is equal to p/_3_. The volumes of the embryos elliptical in their cross section were calculated by using the formula:

V_e_ = ^4^/_3_pab^2^

where a is ½ of embryo length, b is ½ of embryo width, and ^4^/_3_p is equal to 4.188. Percentage air space was calculated by using the formula: [(V_t_-V_e_)/V_t_] .100.

## Results and discussion

**Figure 1.**
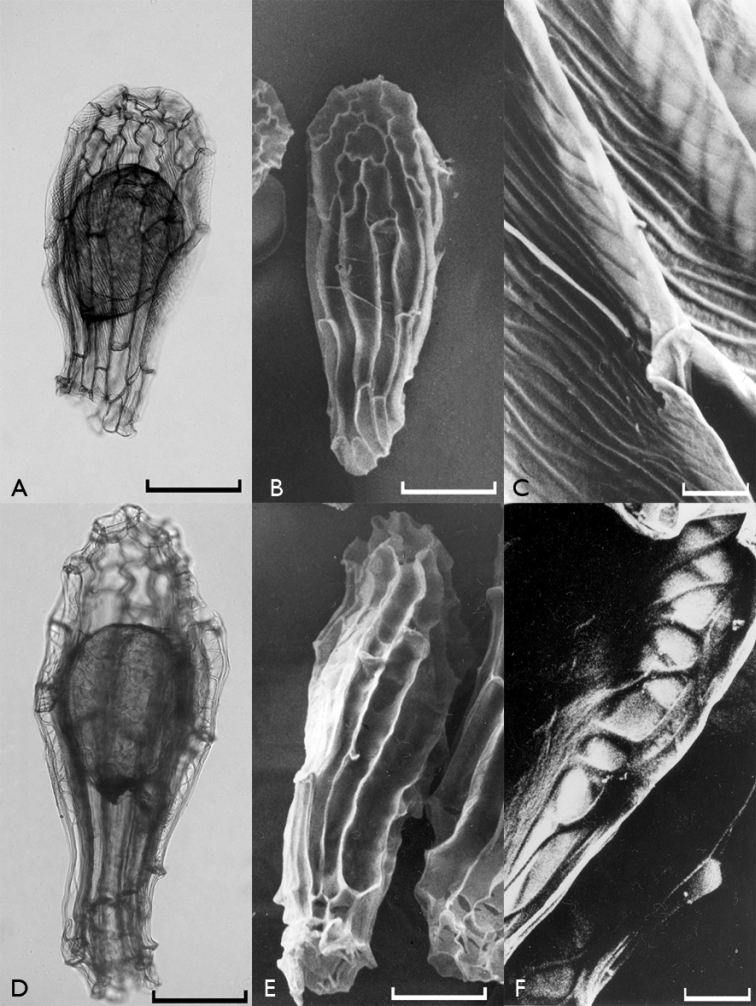
Light microscope (**A**, **D**) and scanning electron microscope (**B**, **C**, **E**, **F**) photographs of *Anacamptis coriophora* (**A**, **B**, **C**) and Anacamptis laxiflora subsp. laxiflora (**D**, **E**, **F**) seeds. Scale bars: 0.1 mm (**A**, **B**, **D**, **E**) and 0.01 mm (**C**, **F**).

**Figure 2.**
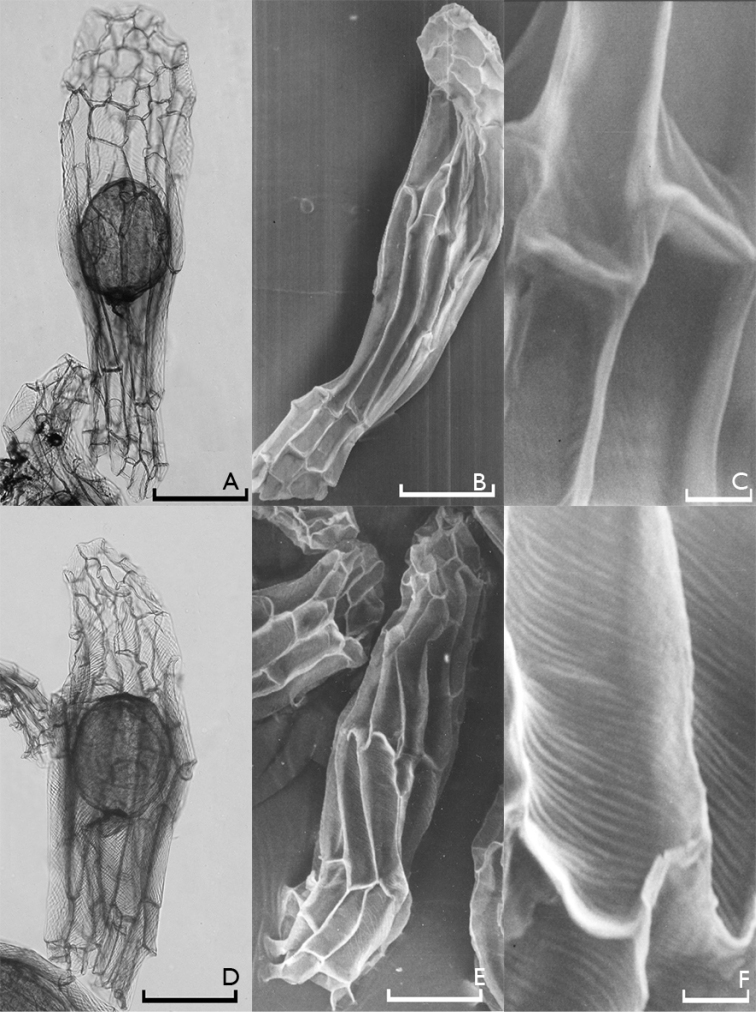
Light microscope (**A**, **D**) and scanning electron microscope (**B**, **C**, **E**, **F**) photographs of Anacamptis morio subsp. morio (**A**, **B**, **C**) and *Anacamptis papilionacea* (**D**, **E**, **F**) seeds. Scale bars: 0.1 mm (**A**, **B**, **D**, **E**) and 0.01 mm (**C**, **F**).

**Figure 3.**
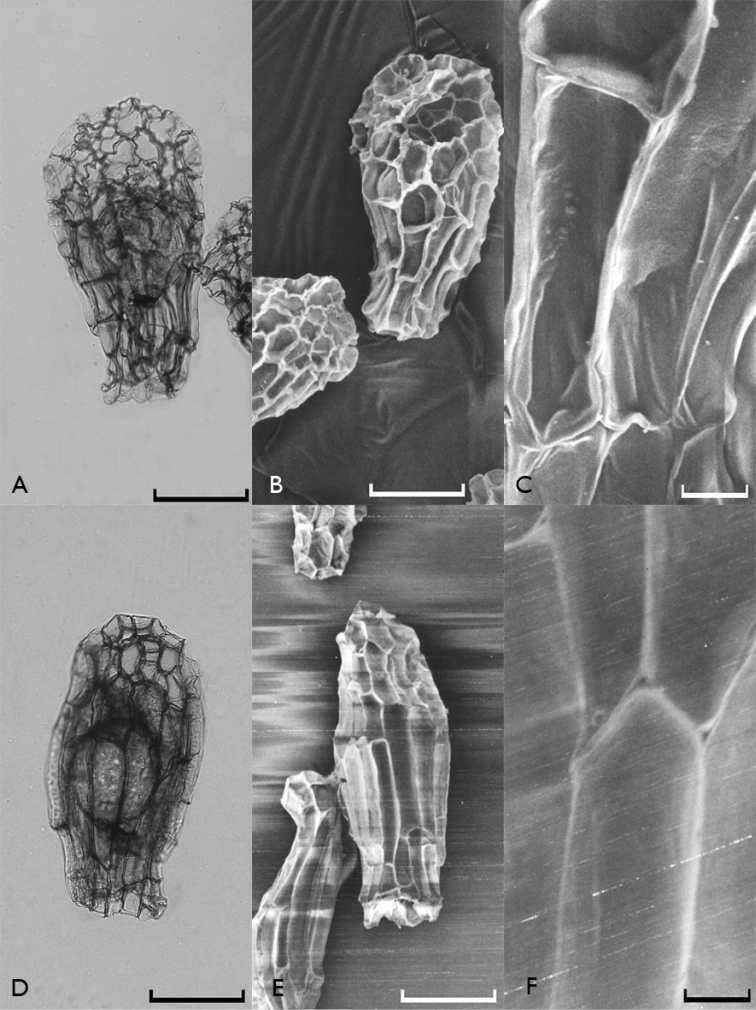
Light microscope (**A**, **D**) and scanning electron microscope (**B**, **C**, **E**, **F**) photographs of Orchis mascula subsp. mascula (**A**, **B**, **C**) and Orchis purpurea subsp. purpurea (**D**, **E**, **F**) seeds. Scale bars: 0.1 mm (**A**, **B**, **D**, **E**) and 0.01 mm (**C**, **F**).

**Figure 4.**
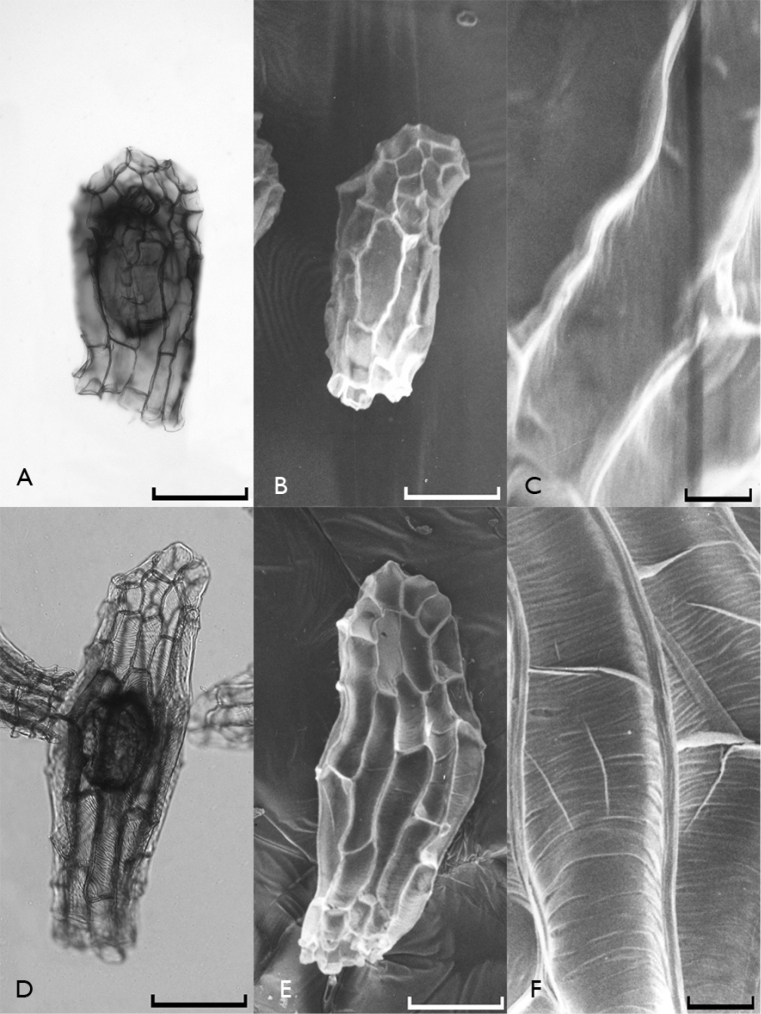
Light microscope (**A**, **D**) and scanning electron microscope (**B**, **C**, **E**, **F**) photographs of Orchis simia subsp. simia (**A**, **B**, **C**) and Neotinea tridentata subsp. tridentata (**D**, **E**, **F**) seeds. Scale bars: 0.1 mm (**A**, **B**, **D**, **E**) and 0.01 mm (**C**, **F**).

All investigated orchid seeds were fusiform in shape and had transparent and elliptical embryos (Figures 1–4). Their testae colors were different shades of brown. The measurements of the seeds as revealed by light microscopy investigations are given in Table 2.

**Table 2.** Measurement data of orchid seeds and embryos.

Species	EDTU	Figure	Embryos			Seeds			Vs/Ve (mm3 x10-3)	Percent Air Space
			L/S.D. (mm)	W/S.D. (mm)	L/W	L/S.D. (mm)	W/S.D. (mm)	L/W		
*Anacamptis coriophora*	6075	1A−1C	0.177/0.015	0.116/0.014	1.532	0.397/0.040	0.186/0.021	2.137	3.59/1.24	65.60
*Anacamptis laxiflora*	6074	1D−1F	0.225/0.039	0.140/0.015	1.606	0.599/0.097	0.208/0.022	2.880	6.78/2.31	65.98
Anacamptis morio subsp. morio	6056		0.124/0.011	0.092/0.011	1.351	0.400/0.051	0.142/0.007	2.824	2.10/0.54	74.19
Anacamptis morio subsp. morio	6058		0.137/0.015	0.097/0.007	1.413	0.420/0.053	0.143/0.089	2.927	2.26/0.68	70.11
Anacamptis morio subsp. morio	6059		0.158/0.019	0.118/0.018	1.339	0.376/0.045	0.171/0.024	2.191	2.89/1.15	60.34
Anacamptis morio subsp. morio	6062		0.173/0.020	0.129/0.014	1.344	0.513/0.037	0.161/0.018	3.196	3.46/1.50	56.82
Anacamptis morio subsp. morio	6063		0.156/0.026	0.097/0.017	1.609	0.640/0.067	0.152/0.017	4.197	3.89/0.76	80.41
Anacamptis morio subsp. morio	6065	2A−2C	0.152/0.012	0.115/0.011	1.319	0.506/0.068	0.147/0.019	3.435	2.87/1.05	63.38
Anacamptis morio subsp. morio	6067		0.142/0.019	0.106/0.017	1.335	0.452/0.031	0.141/0.023	3.209	2.34/0.83	64.50
Anacamptis morio subsp. morio	6265		0.183/0.014	0.128/0.011	1.425	0.573/0.095	0.177/0.023	3.247	4.67/1.57	66.43
Anacamptis morio subsp. morio	6267		0.160/0.026	0.106/0.012	1.517	0.503/0.077	0.148/0.018	3.398	2.88/0.93	67.60
Average for *Anacamptis morio*			0.157	0.103	1.526	0.482	0.156	3.096	3.08/1.07	65.14
*Anacamptis papilionacea*	6079	2D−2F	0.138/0.027	0.104/0.022	1.327	0.451/0.076	0.162/0.027	2.778	3.11/0.78	74.84
Orchis mascula subsp. mascula	6132	3A−3C	0.124/0.016	0.104/0.016	1.191	0.326/0.035	0.195/0.032	1.674	3.24/0.70	78.32
Orchis purpurea subsp. purpurea	6103	3D−3F	0.138/0.022	0.086/0.012	1.602	0.450/0.030	0.144/0.017	3.119	2.45/0.53	78.21
Orchis purpurea subsp. purpurea	6110		0.119/0.016	0.086/0.009	1.381	0.356/0.082	0.142/0.018	2.514	1.87/0.46	75.30
Orchis purpurea subsp. purpurea	6116		0.118/0.014	0.079/0.008	1.484	0.263/0.026	0.118/0.012	2.221	0.96/0.39	59.50
Orchis purpurea subsp. purpurea	6119		0.143/0.016	0.098/0.008	1.461	0.480/0.042	0.166/0.014	2.902	3.44/0.72	79.16
Average for *Orchis purpurea*			0.129	0.111	1.169	0.387	0.142	2.719	2.18/0.53	75.90
Orchis simia subsp. simia	6080	4A−4C	0.148/0.017	0.093/0.015	1.593	0.357/0.029	0.166/0.022	2.147	2.58/0.67	73.92
Neotinea tridentata subsp. tridentata	6092		0.158/0.024	0.125/0.024	1.260	0.578/0.075	0.185/0.025	3.117	5.19/1.29	75.12
Neotinea tridentata subsp. tridentata	6120	4D−4F	0.145/0.016	0.101/0.012	1.428	0.448/0.050	0.157/0.029	2.865	2.87/0.78	72.96
Neotinea tridentata subsp. tridentata	6136		0.157/0.013	0.104/0.013	1.511	0.449/0.044	0.153/0.017	2.942	2.73/0.89	67.24
Average for *Neotinea tridentata*			0.153	0.110	1.391	0.492	0.165	2.983	3.60/0.99	72.55
Average for orchids studied			0.151	0.106	1.430	0.454	0.160	2.853	3.152/0.942	70.00

* S.D. standard deviation

When testae and embryos were investigated for their colors, the following patterns were obtained: Orchis mascula subsp. mascula and Anacamptis laxiflora subsp. laxiflora were light brown, *Anacamptis coriophora*, Anacamptis morio subsp. morio and *Anacamptis papilionacea* were brown, Orchis purpurea subsp. purpurea and Neotinea tridentata subsp. tridentata were dark brown and Orchis simia subsp. simia was darker brown than the rest.

It is possible to divide the orchid species found in Edirne into two groups according to their testa morphologies. The first group includes *Anacamptis coriophora* (Fig. 1A–1C), Anacamptis laxiflora subsp. laxiflora (Fig. 1D–1F), Anacamptis morio subsp. morio (Fig. 2A–2C), *Anacamptis papilionacea* (Fig. 2D–2F) and Neotinea tridentata subsp. tridentata (Fig. 4D–4F) which are the taxa whose anticlinal and periclinal walls of testa cells have reticulations. The second group consists of Orchis mascula subsp. mascula (Fig. 3A–3C), Orchis purpurea subsp. purpurea (Fig. 3D–3F) and Orchis simia subsp. simia (Fig. 4A–4C) whose their testa cell walls are smooth and without reticulations.

When the reticulations were analyzed, it appeared that they showed minute anastomosis. Some orchids, especially the tropical ones, have conspicuous reticulations such as *Calypso bulbosa* (L.) Oakes (Arditti and Ghani 2000), but this was not the case in the Turkish species we included in the present study. Reticulation directions showed differences among species. It was more or less transverse in Neotinea tridentata subsp. tridentate (Fig. 4f), diagonal in *Anacamptis coriophora* (Fig. 1C) and longitudinally diagonal in *Anacamptis papilionacea* (Fig. 2F). Reticulations in these species were conspicuous particularly in their periclinal walls. On the other hand, reticulations in Anacamptis morio subsp. morio were inconspicuous since they were thin and transversely diagonal (Fig. 2C). Testa cells of Anacamptis laxiflora subsp. laxiflora appeared to be different from those of the other species. Anticlinal walls of their testa cells were fairly thick and showed unbranched thickenings (Fig. 1F). The periclinal wall investigations showed that the walls were smooth in some species while in some others they had fine reticulations. Additionally, in some seeds, one could barely see fine and inconspicuous reticulations, and then only in basal cells. Testa cell walls of the species with no reticulations generally showed thickenings in their joining regions (Orchis mascula subsp. mascula (Fig. 3C), Orchis purpurea subsp. purpurea (Fig. 3F) and Orchis simia subsp. simia (Fig. 4C)). Among these, folds in periclinal walls could sometimes be observed.

Seed lengths and widths ranged between 0.263–0.640 mm and 0.118–0.208 mm, respectively. The length and width measurements for embryos were 0.118–0.225 mm and 0.079–0.140 mm, respectively. All species are listed in Table 2.

When the mean values of orchid seed morphometry obtained in the present study were compared to those reported in Arditti and Ghani (2000), it appeared that both data were similar. The measurement data given for orchids in Arditti and Ghani (2000) is as follows; testa length 0.49 (± 0.17) mm, width 0.17 (± 0.06) mm and volume 3.93 ± 3.24 mm^3^, embryo length 0.18 (± 0.05) mm, width 0.12 ± 0.04 mm and volume 1.22 (± 0.77) x 10^-3^ mm^3^ and percentage air space 43.01 (± 35.16) mm^3^. When these measurement data are compared to the present findings (Table 2), one can see that they are quite similar and support each other. Similarly, the current morphometric data on *Anacamptis coriophora*, Anacamptis morio subsp. morio, Orchis purpurea subsp. purpurea and Orchis simia subsp. simia was found to be almost identical, with only a few differences, to the ones reported in Arditti and Ghani (2000).

^L^/_W_ ratios provide data on the relative degree of truncation (Arditti 1979). The lowest ^L^/_W_ of 1.674 in Orchis mascula subsp. mascula showed that seeds of this species were the most truncate seeds. This species is followed by Orchis simia subsp. simia, *Anacamptis coriophora* and Orchis purpurea subsp. purpurea with their low ^L^/_W_ ratios implying a high truncate nature. On the other hand, higher ^L^/_W_ values were obtained for *Anacamptis papilionacea*, Anacamptis laxiflora subsp. laxiflora and Neotinea tridentata subsp. tridentata indicating that they have more elongate seeds. The highest ^L^/_W_ ratio of Anacamptis morio subsp. morio seeds (4.197) shows that the seeds of this species are elongate.

The mean lengths and widths of the embryos of the investigated eight taxa were 0.151 mm and 0.106 mm, respectively. The embryos were found to be elliptical with an average ^L^/_W_ value of 1.43. The lowest ^L^/_W_ value of Orchis mascula subsp. mascula led us to conclude that the embryos of this species were sphere-like. This species is followed by *Anacamptis papilionacea*. The high ^L^/_W_ values of the other species is an indication that their embryos are elliptical rather than spherical.

Percentage air space affects the length of time the orchid seeds are in air. Specimens with high percentage air space values are known to spread over longer distances via wind (Arditti 1967, Healey et al. 1980, Chase and Pippen 1988, Kurzweil 1993). The highest percentage air space determined for the seeds investigated ranged from 56% to 80%. Anacamptis morio subsp. morio seeds, a taxon sampled in most of the visited localities, had both the highest and the lowest percentage air space values. The mean air space value for orchid taxa in Edirne province is 70% and Anacamptis morio subsp. morio, *Anacamptis coriophora* and Anacamptis laxiflora subsp. laxiflora were determined to have the lowest value of approximately 65%. Orchis mascula subsp. mascula, on the other hand, whose seeds were short and wide, had the highest mean value of 78%.

As shown in previous studies on orchids, there are a number of diagnostic and phylogenetically informative characters present in orchid seeds. In this study, seed morphologies of eight orchids taxa growing in Edirne province were investigated and criteria that could be used to differentiate the seeds are presented. Also, a key is constructed below, based on seed morphology.

### Identification key of the eight orchid taxa growing in Edirne province

**Table:** 

1	Testa walls reticulate	**2**
–	Testa walls smooth	**6**
2	Reticulations occurring in periclinal walls more or less conspicuous or not at all	**3**
–	Reticulations occurring in periclinal walls conspicuous	**4**
3	Thickenings in anticlinal walls rather conspicuous	**Anacamptis laxiflora subsp. laxiflora**
–	Thickenings in anticlinal walls inconspicuous	**Anacamptis morio subsp. morio**
4	Reticulations in testa cells transversely	**Neotinea tridentata subsp. tridentata**
–	Reticulations different	**5**
5	Reticulations in testa cells transversely diagonal	***Anacamptis coriophora***
–	Reticulations in testa cells longitudinally diagonal	***Anacamptis papilionacea***
6	Seed fusiform	**Orchis purpurea subsp. purpurea**
–	Seed fusiform-oblong	**7**
7	Seed light brown	**Orchis mascula subsp. mascula**
–	Seed dark brown	**Orchis simia subsp. simia**
